# Effects of Preparation Methods on the Structure and Mechanical Properties of Kyanite-Reinforced Alumina Ceramics

**DOI:** 10.3390/nano16070410

**Published:** 2026-03-28

**Authors:** Xuyang Zhang, Qin Wang, Zhuo Wang, Xiufang Wang, Kuilin Lv, Hai-Yan Li

**Affiliations:** 1 China Testing & Certification International Group Co., Ltd., Beijing 100024, China; 2 School of Civil and Transportation Engineering, Beijing University of Civil Engineering and Architecture, Beijing 102616, China

**Keywords:** kyanite-reinforced alumina ceramics, prestress reinforcement, particle enhancement, mechanical properties, microstructure

## Abstract

In this work, kyanite-reinforced alumina ceramics were prepared using the prestress reinforcement method and the particle enhancement method. The effects of different preparation methods on the mechanical properties and microstructures of kyanite-reinforced alumina ceramics were investigated. The results showed that, compared to the pure alumina ceramic, the prestressed alumina ceramic (labeled as P-Al_2_O_3_) prepared by the prestress reinforcement method exhibited a significant improvement (31% higher than that of pure alumina) in flexural strength. This is mainly attributed to the fact that the compressive stress existing on the surface of P-Al_2_O_3_ inhibited crack propagation; therefore, the fracture energy and strength were increased. However, due to the numerous pores and cracks in the fracture surface caused by the decomposition reaction of kyanite, the alumina composites fabricated through the particle enhancement method (labeled C-Al_2_O_3_) displayed lower flexural strength and hardness than those with P-Al_2_O_3_. Additionally, an increase in kyanite content led to a decrease in properties such as flexural strength, Vickers hardness, density, the elastic modulus, and the thermal expansion coefficient, while resulting in an increase in porosity. This work demonstrates the importance of using a suitable preparation method aligned with the specific composite.

## 1. Introduction

Alumina ceramics (Al_2_O_3_) possess a suite of excellent mechanical properties, such as high hardness, an elevated melting point, and robust chemical stability. These attributes underpin their broad utility across various high-tech sectors, including industrial manufacturing, electronics, and aerospace [1,2,3,4]. Nevertheless, the application of Al_2_O_3_ in more diverse fields is hindered by critical material shortcomings, primarily its intrinsic brittleness and pronounced sensitivity to defects. To solve these problems, various methods have been used to improve the mechanical properties of Al_2_O_3_ ceramics [5,6,7], such as improving the purity and density [8,9], using particle-/whisker-/fiber-reinforced composites [10,11,12], prestressed reinforcement [13,14,15], and so on. The particle enhancement method, developed from the principles of dispersion strengthening in metallic materials, involves introducing a second-phase particulate reinforcement into a ceramic matrix to improve its mechanical properties [16]. However, the process of improving the purity and density is complex, expensive, and of limited effectiveness. The particle enhancement method effectively improves the mechanical performance of Al_2_O_3_ ceramic composites. Due to the characteristics of low shrinkage and strong compatibility with alumina, kyanite has been used as a reinforcing material [17]. However, the dispersion, content, and size of the second-phase particles significantly influence the material properties. In contrast, the prestress reinforcement technique offers a more advanced solution due to its straightforward application and marked improvements in both strength and damage tolerance [18,19].

As a typical prestressed strengthening material, tempered glass exhibits strength that is two to five times higher than that of ordinary glass [20,21]. Bao et al. [13] applied prestress reinforcement technology to ceramics through the ion exchange method and by using a coating material with a lower coefficient of thermal expansion on the surfaces of substrates. The results showed that the flexural strength of zirconia (ZrO_2_) ceramics reinforced with Al_2_O_3_ coatings was 45.0% higher than that of pure ZrO_2_ ceramics. The reinforcement method is simple and cost-effective and is suitable for any shape or size. In addition, Jia et al. [22] prepared prestressed ZrO_2_ ceramics coated with mullite and ZrO_2_. The strength and fracture toughness were 39.15% and 26.8% higher than those of pure ZrO_2_ ceramics, respectively. Wu et al. [23] used kyanite as a prestressed coating to strengthen ZrO_2_-reinforced Al_2_O_3_ (ZTA) ceramics. To fabricate prestressed Al_2_O_3_ ceramics, Li et al. [17] employed a kyanite coating. Their work, along with other studies [24,25], demonstrates that this prestressed coating reinforcement method yields a notable enhancement in both the flexural strength and fracture toughness of the ceramic matrix. However, few reports have focused on the comparison of different methods regarding the mechanical strength and microstructures of Al_2_O_3_ ceramics.

In the present work, Al_2_O_3_ ceramics were fabricated separately using two distinct approaches: prestressed coating reinforcement and particle enhancement. The properties, including density, flexural strength, and Young’s modulus, were tested. By comparing the microstructure mechanical properties and the residual stress of the above Al_2_O_3_ ceramics, the strengthening mechanisms were analyzed.

## 2. Experimental Procedure

### 2.1. Materials

Kyanite powder (99.9% purity, China Zhisheng Kuangye Co., Ltd., Xiamen, China) and Al_2_O_3_ powder (Model α, 99.99% purity, Japan Daming Chemical Industry Co., Ltd., Tokyo, Japan) were used as raw materials. To prepare the kyanite coating slurry, ethanol (≥99.97%, Fuyu Reagent Co., Ltd., Beijing, China), polyvinyl butyral (molecular weight 90,000–120,000, Macklin Chemical Reagent Co., Ltd., Shanghai, China), and castor oil (analytical reagent grade, Macklin Chemical Reagent Co., Ltd., Shanghai, China) were used as the solvent, binder, and dispersant, respectively.

### 2.2. Methods

#### 2.2.1. Preparation of P-Al_2_O_3_ by the Prestress Reinforcement Method

**Figure 1:**
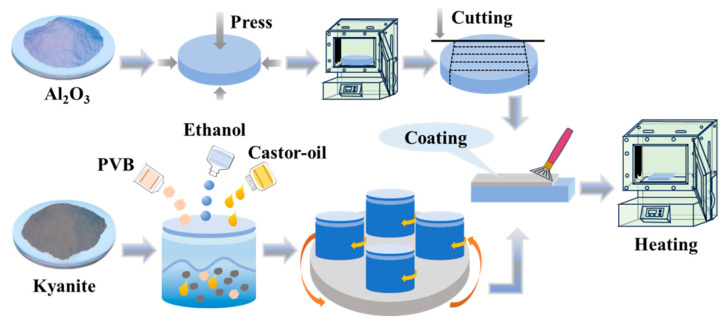
Schematic diagram of P-Al_2_O_3_ preparation process by prestress reinforcement method.

The P-Al_2_O_3_ prepared using the prestressing reinforcement method is shown in Figure 1. The Al_2_O_3_ powder was dry-pressed and pre-fired at 1000 °C for 60 min. Then, it was cut to a size of 3 mm × 4.5 mm × 40 mm. To prepare the coating slurry, a mixture of kyanite powder, ethanol, and castor oil was ball-milled at 300 rpm for 6 h. Then, it was evenly brushed onto both sides of the Al_2_O_3_ substrate. After this, P-Al_2_O_3_ was sintered at 1600 °C for 2 h. The coating thickness could be tailored by adjusting the number of coating applications, the concentration of the slurry, and the polishing process. As measured by optical microscopy, the resulting coating thickness was ≈48 µm [14].

#### 2.2.2. Preparation of C-Al_2_O_3_ by the Particle Enhancement Method

**Figure 2:**
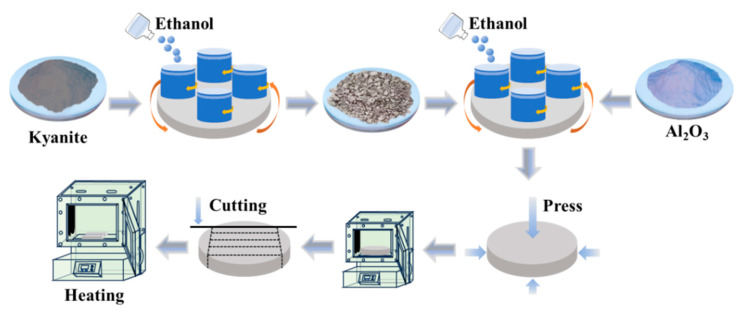
Schematic diagram of C-Al_2_O_3_ preparation process by particle enhancement method.

The C-Al_2_O_3_ prepared by the particle enhancement method is shown in Figure 2. Firstly, kyanite powder was ball-milled at 300 rpm for 24 h. Then, Al_2_O_3_ powder, ethanol, and kyanite powder with mass fractions of 10 wt% (labeled C1), 20 wt% (C2), 30 wt% (C3), 40 wt% (C4), and 50 wt% (C5) were mixed in a ball mill at 300 rpm for 6 h. Then, the mixtures were pressed, cut, and sintered.

### 2.3. Characterization

In this study, the microstructures and compositions of the ceramics were characterized by techniques including X-ray diffraction (XRD, Rigaku SmartLab SE, Tianjin Zhonghuan Co., Ltd., Tianjing, China), X-ray fluorescence (XRF, PANalytical Axios, Tianjin Zhonghuan Co., Ltd., Tianjing, China), and scanning electron microscopy (SEM, ZEISS GeminiSEM 300, Tianjin Zhonghuan Co., Ltd., Tianjing, China). The crystalline phase compositions of the sintered coating and the composite were analyzed using XRD with a scanning angle range of 10° to 80° and a scanning rate of 5°/min. The microstructures and coating thicknesses of the ceramic cross-sections were observed using an SEM instrument equipped with an energy-dispersive spectrometer (EDS). The flexural strength of the samples was characterized by three-point bending tests with a loading speed of 0.5 mm/min and span of 30 mm. Mechanical properties such as hardness, flexural strength, and Young’s modulus were evaluated using a Vickers hardness tester, a universal testing machine, and a dynamic modulus analyzer, respectively. Detailed descriptions of the testing methodologies and parameter ranges are provided in the Supplementary Materials. The data for each mechanical property were averaged over seven tests. The density and porosity of the samples were evaluated using the Archimedes method, in accordance with ASTM C20 [26].

## 3. Results and Discussion

**Figure 3:**
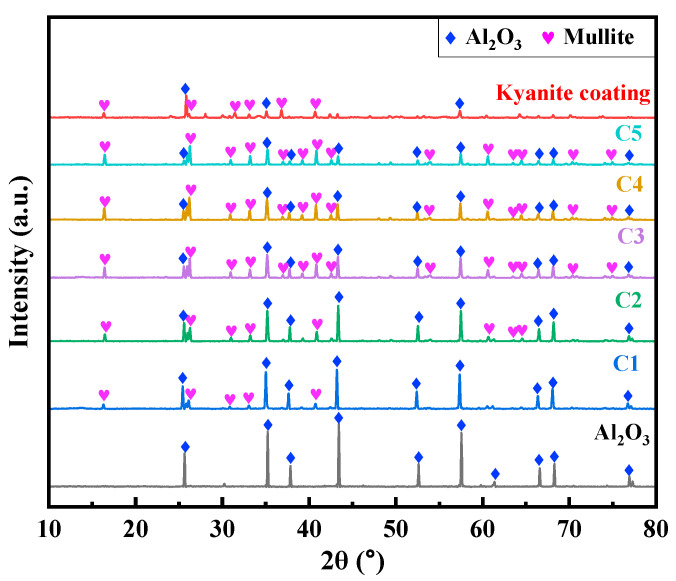
XRD patterns of ceramics prepared in this work.

The XRD results for Al_2_O_3_, P-Al_2_O_3_, and C-Al_2_O_3_ samples are shown in Figure 3. In C-Al_2_O_3_, as the kyanite content increases, the amount of the mullite phase also rises, which is likely to cause excessive expansion and thereby adversely affect its structure. In P-Al_2_O_3_, a mullite phase is also generated in the coating under high-temperature conditions. The reaction equation is as follows:

(1) $$3Al_{2}\mathrm{Si}O_{5}(s)\;⟶\;Al_{6}{\mathrm{Si}}_{2}O_{13}(s)\;+\;\mathrm{Si}O_{2}\;(\mathrm{cristobalite})\;$$

(2) $$3Al_{2}O_{3}(s)+2\mathrm{Si}O_{2}\;(\mathrm{cristobalite})\;⟶\;Al_{6}{\mathrm{Si}}_{2}O_{13}\left(s\right)$$

**Figure 4:**
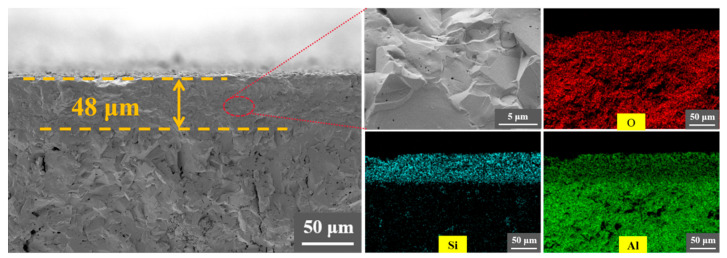
SEM fractographs and corresponding EDS maps of the cross-sections for P-Al_2_O_3_ samples.

**Figure 5:**
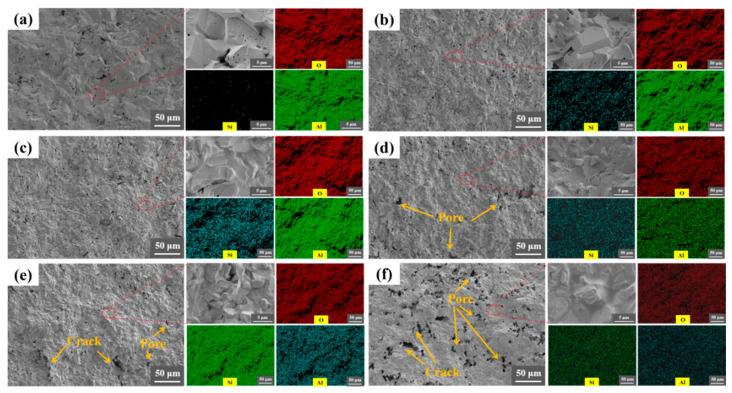
SEM fractographs and corresponding EDS maps of the cross-sections for ceramic samples: (**a**) Al_2_O_3_; (**b**) C-Al_2_O_3_ (labeled C1); (**c**) C-Al_2_O_3_ (C2); (**d**) C-Al_2_O_3_ (C3); (**e**) C-Al_2_O_3_ (C4); (**f**) C-Al_2_O_3_ (C5).

Figure 4 and Figure 5 show the fracture surfaces of the Al_2_O_3_ ceramics prepared by different methods. The fracture morphology reveals a primarily transgranular fracture mode. As shown in Figure 4, the thickness of the coating in P-Al_2_O_3_ is ≈48 μm. No cracks or interfacial debonding are observed at the interface, indicating strong interfacial adhesion. This is attributed to the in situ reaction shown in Equations (1) and (2), which is beneficial to improve the adhesion strength of the interface.

As shown in Figure 5, the Al_2_O_3_ ceramics exhibit a high density with relatively few pores on the fracture surface. For the C-Al_2_O_3_ composite, EDS elemental mapping indicates that the generated mullite particles are uniformly dispersed within the Al_2_O_3_ substrate. As shown in the SEM images, both the size and number of pores on the fracture surface of C-Al_2_O_3_ gradually increase with rising kyanite content. In particular, as the content of kyanite reaches 50% (Figure 5f), extensive pores and cracks are introduced because of the decomposition of kyanite.

**Figure 6:**
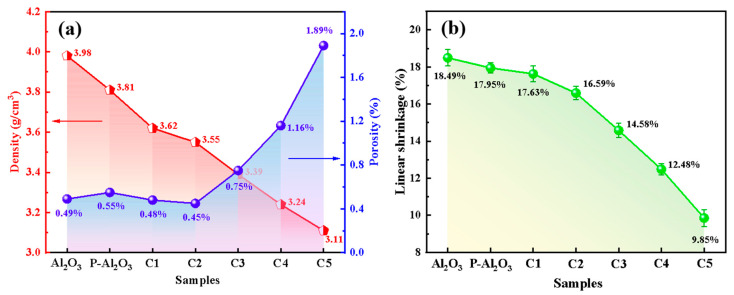
Density (**a**), porosity (**a**), and linear shrinkage rate (**b**) of each group of Al_2_O_3_ ceramic samples.

The sample density and porosity were assessed via Archimedes’ principle, with the corresponding results presented in Figure 6a. The density and porosity of pure Al_2_O_3_ ceramics are 3.98 g/cm^3^ and 0.49%, respectively. However, the density of the Al_2_O_3_-based composites decreased. In particular, in C-Al_2_O_3_, the generated mullite content increased with rising kyanite addition, which consequently led to a decrease in the density of the composite ceramics and an increase in porosity. Figure 6b illustrates the linear shrinkage of each group of samples after sintering at 1600 °C. In sum, as the kyanite content increased, the linear shrinkage of the composite decreased.

**Figure 7:**
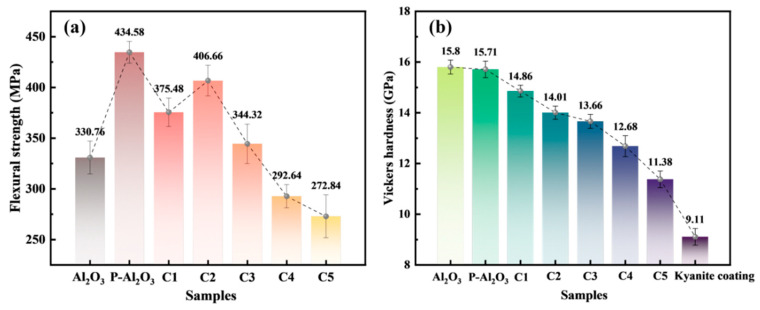
Flexural strength (**a**) and Vickers hardness (**b**) of different ceramic samples.

The flexural strength of samples was measured by three-point bending tests. As shown in Figure 7a, the measured flexural strength of P-Al_2_O_3_ was 434.58 ± 10.69 MPa, which was 31% higher than that of the pure Al_2_O_3_ ceramic (330.76 ± 16.18 MPa). The large improvement in flexural strength is due to the compressive stress on the surface of P-Al_2_O_3_, which hinders rapid crack propagation [27]. Figure 7b presents the Vickers hardness of the samples. As shown in this figure, the pure Al_2_O_3_ ceramic and P-Al_2_O_3_ possess equivalent hardness. However, the Vickers hardness of C-Al_2_O_3_ gradually decreases as the kyanite content increases. The pinning effect initially stabilizes or slightly improves the strength at low kyanite content. However, the enhancement effect decreased because of the increased porosity as the content of kyanite increased. As the content of kyanite increased sequentially, more defects caused by the decomposition of kyanite were introduced; then, obvious reductions in flexural strength and hardness appeared. A Weibull analysis was performed on specimens fabricated by the above two methods, as shown in Figure S5. Compared to C2 specimens prepared by particle enhancement, the P-Al_2_O_3_ ceramics fabricated via the prestress reinforcement method exhibit higher strength and reliability. Additionally, the fracture toughness of the samples is shown in Figure S6. As illustrated in Figure S6, P-Al_2_O_3_ exhibited the highest fracture toughness of 7.10 Mpa·m^1/2^, as compared to C-Al_2_O_3_ and Al_2_O_3_ ceramics. It has been proven that the prestress reinforcement method improves the mechanical strength and fracture toughness of Al_2_O_3_ ceramics.

**Figure 8:**
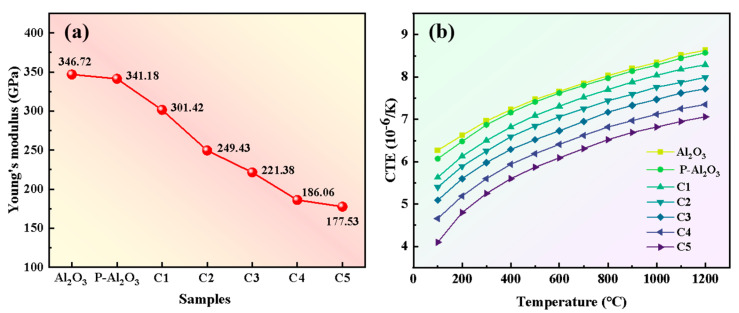
Young’s moduli (**a**) and thermal expansion coefficients (**b**) of each group of Al_2_O_3_ ceramic samples.

To clarify the prestress strengthening mechanism, the residual stress in P-Al_2_O_3_ was calculated. According to ISO 20343 [28] and ISO 23458 [29], residual stress is related to the Young’s modulus and coefficient of thermal expansion (CTE). As shown in Figure 8a,b, both the Young’s modulus and CTE of the alumina-based composites decreased as the content of mullite increased. This is attributed to the fact that the transformation of kyanite into mullite caused a greater increase in porosity. To better clarify the prestress mechanism, the residual compressive stress within the coating was analyzed and calculated using the relative method. Based on Equations S(1)–S(3), the residual compressive stress of the coating was calculated to be 1120 MPa. The relevant parameters used to calculate the residual stress are summarized in Table S1. Under external loading, this stress can partially offset tensile stresses, suppress crack propagation, and enhance flexural strength. Above all, the prestress reinforcement method is more suitable for the fabrication of Al_2_O_3_-based ceramics with high strength.

## 4. Conclusions

In this study, the prestress reinforcement method and the particle enhancement method were used to prepare kyanite and Al_2_O_3_ materials, marked as P-Al_2_O_3_ and C-Al_2_O_3_, respectively. The microstructures and mechanical properties of the above samples were investigated. The results show the following:

1. The strengthening mechanism of P-Al_2_O_3_ is attributed to the compressive stress on the surface, which can inhibit the initiation and propagation of cracks. Thus, more fracture energy is needed to break the samples.
2. The strengthening mechanism of C-Al_2_O_3_ consists of the pinning effect. However, the higher the content of kyanite, the higher the porosity and the lower the strength of P-Al_2_O_3_.
3. In this work, the flexural strength and fracture toughness of P-Al_2_O_3_ were much higher than those of C-Al_2_O_3_.
4. The prestress reinforcement method is a simple, cost-effective, and effective approach for the fabrication of kyanite-reinforced Al_2_O_3_ ceramics with high strength and high fracture toughness.

## Data Availability

The original contributions presented in this study are included in the article and Supplementary Materials. Further inquiries can be directed to the corresponding author.

## Supplementary Materials

The following supporting information can be downloaded at https://www.mdpi.com/article/10.3390/nano16070410/s1. Figure S1: Composition of the kyanite in XRF analysis, Figure S2: Schematic diagram of prestressed alumina ceramics, Figure S3: Particle size distribution of kyanite powder, Figure S4: Particle size distribution of kyanite powder after 24 h ball milling, Figure S5: Weibull analysis of P-Al_2_O_3_ and C-Al_2_O_3_(C2), Figure S6: Fracture toughness analysis of P-Al_2_O_3_ and C-Al_2_O_3_; Table S1: Calculation results of residual compressive stress of prestressed coating of kyanite.

## References

[1] Ghorbanhossaini A., Rafiee R., Pligovka A., Salerno M. (2023). Dental composites with strength after aging improved by using anodic nanoporous fillers: Experimental results, modeling, and simulations. Eng. Comput..

[2] Kim T.G., Raju K., Lee H.K. (2021). Pressure-less joining of alumina ceramics by the reaction-bonded aluminum oxide (RBAO) method. J. Eur. Ceram. Soc..

[3] Zhao J., Wang L., Mao X., An L., Liu Y., Wang S., Zhang J., Feng K. (2022). Preparation and properties of porous alumina ceramics for ultra-precision aerostatic bearings. Ceram. Int..

[4] Kohama K. (2020). Joining of alumina ceramics using silicon–magnesium composite filler for high-temperature applications. Sci. Technol. Weld. Join..

[5] Kaur K., Talibi M., Parmar H. (2022). Do you know your ceramics? Part 4: Alumina. Br. Dent. J..

[6] Zhu C., Feng C., Yang X., Peng Z. (2020). Effects of sintering temperature on mechanical properties of alumina fiber reinforced alumina matrix composites. J. Sol-Gel Sci. Technol..

[7] Szutkowska M., Cyboroń J., Podsiadło M., Polczyk T. (2022). Residual stresses in alumina matrix composites reinforced with Ti (C, N). Ceram. Int..

[8] Zhao D., Bi G., Chen J., Zhu J., Niu F., Ma G., Wu D. (2024). Melt-grown behaviour of heat treated high-purity alumina ceramics prepared by laser directed energy deposition. Ceram. Int..

[9] Xu H., Zou J., Crookes R., Ke B., Li Y., Zhu Q., Wang Q., Zhang J., Wang W., Ji W. (2024). Enhanced properties of nanocrystalline alumina ceramic with compressive prestress and coherently aligned nanograins. J. Am. Ceram. Soc..

[10] Tovar-Vargas D., Turon-Vinas M., Anglada M., Jimenez-Pique E. (2020). Enhancement of mechanical properties of ceria-calcia stabilized zirconia by alumina reinforcement. J. Eur. Ceram. Soc..

[11] Zheng W., Wu J.M., Chen S., Yu K., Zhang J., Shi Y. (2022). Improved mechanical properties of SiC fiber reinforced silica-based ceramic cores fabricated by stereolithography. J. Mater. Sci. Technol..

[12] Chen R., Bratten A., Rittenhouse J., Leu M.C., Wen H. (2023). Additive manufacturing of continuous carbon fiber-reinforced SiC ceramic composite with multiple fiber bundles by an extrusion-based technique. Ceram. Int..

[13] Bao Y., Kuang F., Sun Y., Li Y., Wan D., Shen Z., Ma D., He L. (2019). A simple way to make pre-stressed ceramics with high strength. J. Mater..

[14] Cao D., Lv K., Bao Y., Tian Y., Wan D. (2023). Thickness effect of an alumina–zirconia–mullite composite coating on the properties of zirconia. RSC Adv..

[15] Fu S., Jia Z., Ding W., Bao Y., Wan D. (2024). Synthesis and characterization of a high-strength alumina ceramic reinforced by AlN-Al_2_O_3_ coating. J. Mater. Sci..

[16] Duan G., Sakai M. (2022). An enhanced semi-implicit particle method for simulating the flow of droplets with free surfaces. Comput. Methods Appl. Mech. Eng..

[17] Li H.Y., Hao H., Tian Y., Liu X.-G., Wan D., Bao Y. (2022). Temperature dependence of flexural strength and residual stress of Al_2_O_3_ reinforced by kyanite coating. Ceram. Int..

[18] Nawy E.G. (1996). Prestressed Concrete. A Fundamental Approach.

[19] Pokorný P., Chobotský T., Prodanovic N., Steinerová V., Hurtig K. (2024). Bond Strength and Corrosion Protection Properties of Hot-Dip Galvanized Prestressing Reinforcement in Normal-Strength Concrete. J. Compos. Sci..

[20] Belenky A., Rittel D. (2012). Static and dynamic flexural strength of 99.5% alumina: Relation to porosity. Mech. Mater..

[21] Xu H.M., Wang X.B., Wang B.C., Xue Y., Qi Y., Kong L. (2023). Study of the Influence Factors for Ballistic Performance of Glass Fiber Reinforced Composites. J. Phys. Conf. Ser..

[22] Jia Z.J., Fu S., Li H.Y., Bao Y.W., Zhang C., Wan D.T. (2024). Enhanced Fracture Strength and Toughness of Zirconia by Coating the Pre-Stressed Mullite-Zirconia. J. Ceram. Sci. Technol..

[23] Wu H.L., Li H., Cao D., Qiu Y., Wan D., Bao Y. (2023). The Effects of Compressive Residual Stress on Properties of Kyanite-Coated Zirconia Toughened Alumina Ceramics. Materials.

[24] Hao H., Li H., Wan D., Bao Y. (2022). Enhanced flexural strength and thermal shock resistance of alumina ceramics by mullite/alumina pre-stressed coating. J. Inorg. Mater..

[25] Li H.Y., Wu H., Han Y., Liu X., Wan D., Bao Y. (2024). Enhanced high temperature properties of ZTA prestressed ceramics reinforced by cordierite coating. Int. J. Appl. Ceram. Technol..

[26] (2022). Standard Test Methods for Apparent Porosity, Water Absorption, Apparent Specific Gravity, and Bulk Density of Burned Refractory Brick and Shapes by Boiling Water.

[27] Zhang X., Li Y., Sun Y., Hao D., Li K., Wan D., Bao Y. (2022). Improving the flexural strength of porcelain by residual stress in anorthite coating. Int. J. Appl. Ceram. Technol..

[28] (2017). Fine Ceramics (Advanced Ceramics, Advanced Technical Ceramics)—Test Method for Determining Elastic Modulus of Thick Ceramic Coatings at Elevated Temperature.

[29] (2020). Fine Ceramics (Advanced Ceramics, Advanced Technical Ceramics)—Test Method for Determining Thermal Expansion Coefficient and Residual Stress of CVD Ceramic Coatings.

