# Supplementary material for: Effects of Preparation Methods on the Structure and Mechanical Properties of Kyanite-Reinforced Alumina Ceramics
Source: Nanomaterials (Basel). 2026 Mar 28;16(7):410. doi: 10.3390/nano16070410 (PMC13074533; doi:10.3390/nano16070410)
Supplement: Supplementary file 1 [file nanomaterials-16-00410-s001.zip › nanomaterials-4152499-supplementary.pdf]

Supporting Materials

Effects of Preparation Methods on the Structure and Mechanical Properties of Kyanite-Reinforced Alumina Ceramics

Xuyang Zhang [1,2], Qin Wang [2], Zhuo Wang [2], Xiufang Wang [2], Kuilin Lv [1] and Hai-Yan Li [1,*]

[1] China Testing & Certification International Group Co., Ltd., Beijing 100024, China;
[2] School of Civil and Transportation Engineering, Beijing University of Civil Engineering and Architecture, Beijing 102616, China
* Correspondence: lihaiyan@ctc.ac.cn

2. Experimental procedure

2.2. Methods

2.2.1. Preparation of prestressed alumina ceramics by the coating method

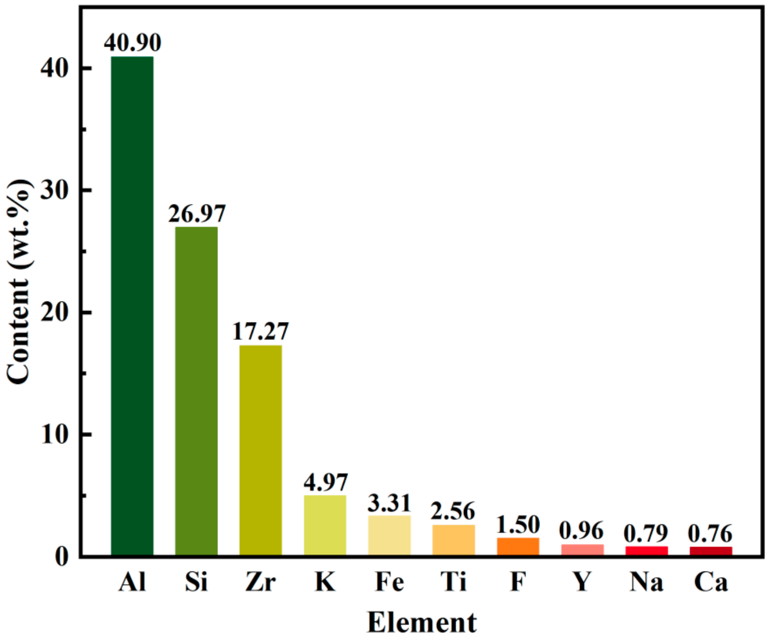

Figure S1. Composition of the kyanite in XRF analysis.

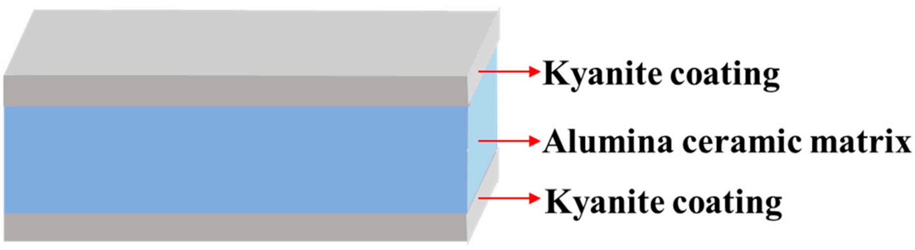

Figure S2. Schematic diagram of prestressed alumina ceramics.

2.2.2. Alumina composite ceramics were prepared by particle toughening method

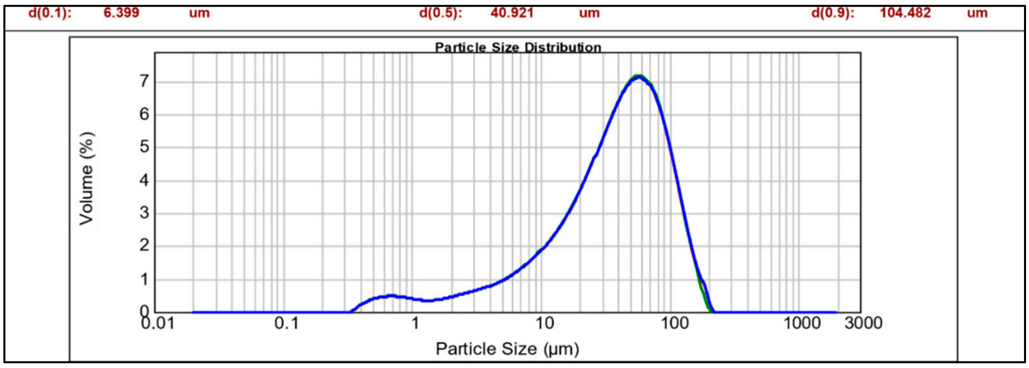

Figure S3. Particle size distribution of kyanite powder.

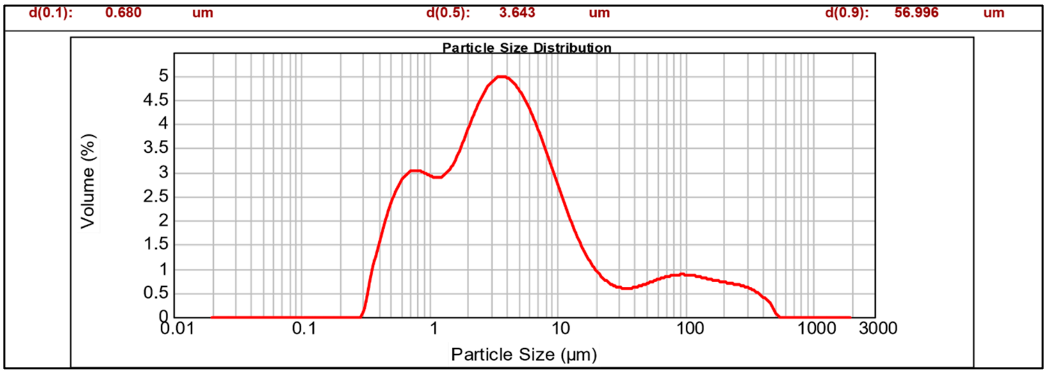

Figure S4. Particle size distribution of kyanite powder after 24 h ball milling.

2.3. Characterization

The crystalline phase composition of the sintered coating and the composite was analyzed using X-ray Diffraction (XRD, Rigaku SmartLab SE, Japan) with a scanning angle range of 10° to 80° and a scanning rate of 5°/min. The elemental composition of the kyanite was analyzed using an X-ray Fluorescence Spectrometer (XRF, ARL PERFORM'X, Thermo Scientific). The microstructure and coating thickness of the ceramic cross-sections were observed using a scanning electron microscope (SEM, ZEISS GeminiSEM 300, Germany) equipped with an energy dispersive spectrometer (EDS). The density and porosity were measured using the Archimedes' principle method. The flexural strength of the samples was characterized by three-point bending test with the loading speed of 0.5 mm/min. The Vickers hardness of the ceramics was measured using a Tukon2500B hardness tester (Wilson, America) with a test force of 1000 gf and a dwell time of 15 s. The Young's modulus of the samples was tested using a solid material Young's modulus tester developed by the China Building Materials Test & Certification Center. The thermal expansion coefficient of the samples within the temperature range of 100 °C to 1200 °C was measured by using a Thermal Expansion Analyzer (DIL 402C, Linseis, Germany).

3. Results and discussion

To better elucidate the mechanism of kyanite coating-enhanced alumina ceramics, the residual compressive stress within the coating was analyzed and calculated using the relative method. Based on Equations (S1), (S2), and (S3) [1,2], the residual compressive stress of the coating was calculated to be 1120 MPa. The relevant results are summarized in Table S1.

Previous studies have shown that when the temperature exceeds 1100°C, the flexural strength of prestressed alumina ceramics tends to converge with that of alumina ceramics

without coating enhancement. This indicates that at high temperatures, the residual compressive stress within the coating gradually relaxes, leading to a reduction in the enhancement effect. Based on this finding, 1100°C was set as the reference temperature difference in Formula (S2).

$$\alpha_c = \alpha - \frac{E_s S_s}{E_c S_c} (\alpha_s - \alpha) \quad \text{(S1)}$$

$$\sigma_t = E_s \cdot \alpha_s \cdot \Delta T \quad \text{(S2)}$$

$$\sigma_c = \left(\frac{S_s}{S_c}\right) \cdot \left\{ 1 - \frac{\left[\frac{E_s S_s}{E_c S_c} + \frac{\alpha_c}{\alpha_s}\right]}{\left[1 + \frac{E_s S_s}{E_c S_c}\right]} \right\} \sigma_t \quad \text{(S3)}$$

Here, α, α_c, and α_s denote the coefficients of thermal expansion for the prestressed alumina ceramic, the coating, and the substrate, respectively. E_c and E_s represent the Young's moduli of the coating and the substrate, respectively. h_c and h_s refer to the thicknesses of the coating and the substrate; σ_c is the residual compressive stress; σ_t stands for the thermal stress; and ΔT indicates the temperature difference.

Table S1. Calculation results of residual compressive stress of prestressed coating of kyanite.

h_c/mm	h_s/mm	E_c/GPa	E_s/GPa	α_c/10^{-6}K^{-1}	α_s/10^{-6}K^{-1}	ΔT/°C	σ_c/MPa
0.048	2.350	251.199	346.720	4.515	8.630	1100	1120.339

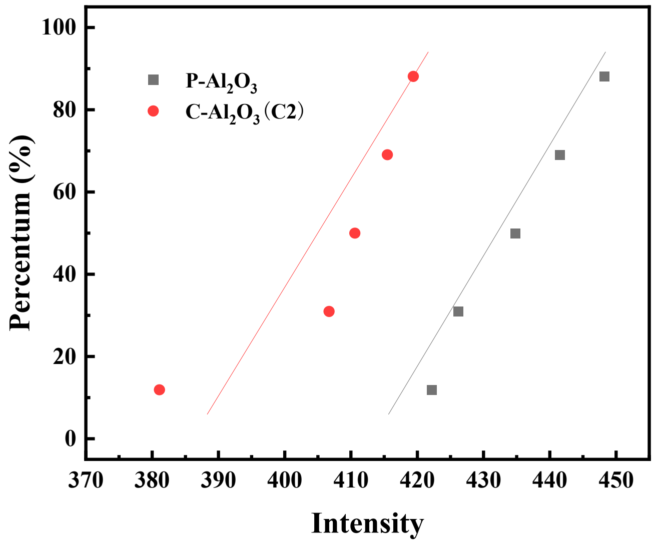

Figure S5. Weibull analysis of P-Al_2O_3 and C-Al_2O_3(C2)

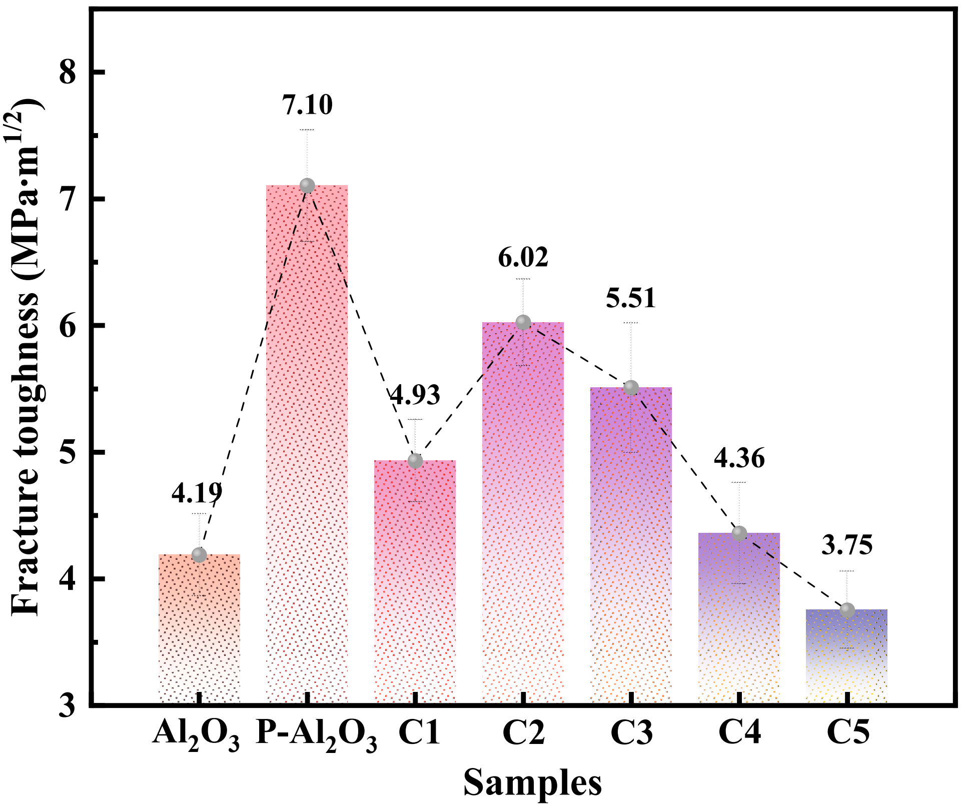

Figure S6. Fracture toughness analysis of P-Al_2O_3 and C-Al_2O_3

References

1. Technical Committee ISO/TC 206-Fine ceramics. ISO 23458:2020 Fine ceramics (advanced ceramics, advanced technical ceramics)-Test method for determining thermal expansion coefficient and residual stress of CVD ceramic coatings[S]. Geneva, Switzerland, 2020.
2. Technical Committee ISO/TC 206-Fine ceramics. ISO 20343:2017 Fine ceramics (advanced ceramics, advanced technical ceramics)-Test method for determining elastic modulus of thick ceramic coatings at elevated temperature[S]. Geneva, Switzerland, 2017.
